# Nipple Pain in Breastfeeding Mothers: Incidence, Causes and Treatments

**DOI:** 10.3390/ijerph121012247

**Published:** 2015-09-29

**Authors:** Jacqueline C. Kent, Elizabeth Ashton, Catherine M. Hardwick, Marnie K. Rowan, Elisa S. Chia, Kyle A. Fairclough, Lalitha L. Menon, Courtney Scott, Georgia Mather-McCaw, Katherine Navarro, Donna T. Geddes

**Affiliations:** 1 School of Chemistry & Biochemistry, The University of Western Australia, 35 Stirling Highway, Crawley WA 6009, Australia; E-Mails: Marnie.Rowan@uwa.edu.au (M.K.R.); Donna.Geddes@uwa.edu.au (D.T.G.); 2 Breast Feeding Centre of WA, King Edward Memorial Hospital for Women, 374 Bagot Rd, Subiaco WA 6008, Australia; E-Mails: Liz.Ashton@health.wa.gov.au (E.A.); CatherineMeria.Hardwick@health.wa.gov.au (C.M.H.); 3 School of Medicine and Pharmacology, The University of Western Australia, 35 Stirling Highway, Crawley WA 6009, Australia; E-Mails: Elisa.Chia@health.wa.gov.au (E.S.C.); fairck01@gmail.com (K.A.F.); Lalitha.Menon@health.wa.gov.au (L.L.M.); cscott9294@gmail.com (C.S.); 20930736@student.uwa.edu.au (G.M.-M.); 20953365@student.uwa.edu.au (K.N.)

**Keywords:** breastfeeding, nipple pain, treatments, diagnosis

## Abstract

*Background*: Persistent nipple pain is one of the most common reasons given by mothers for ceasing exclusive breastfeeding. We aimed to determine the frequency of nipple pain as a reason for consultation, the most common attributed aetiologies, and the effectiveness of the advice and treatment given. *Methods*: All consultations at the Breast Feeding Centre of Western Australia (WA) were audited over two six-month periods in 2011 (*n* = 469) and 2014 (*n* = 708). Attributed cause(s) of nipple pain, microbiology results, treatment(s) advised, and resolution of pain were recorded. *Results*: Nipple pain was one of the reasons for consultation in 36% of cases. The most common attributed cause of nipple pain was incorrect positioning and attachment, followed by tongue tie, infection, palatal anomaly, flat or inverted nipples, mastitis, and vasospasm. Advice included correction of positioning and attachment, use of a nipple shield, resting the nipples and expressing breastmilk, frenotomy, oral antibiotics, topical treatments, and cold or warm compresses. Pain was resolving or resolved in 57% of cases after 18 days (range 2–110). *Conclusion*: The multiple attributed causes of nipple pain, possibly as a result of a cascade of events, suggests that effective early lactation management for prevention of nipple pain and early diagnosis and effective treatment are crucial to avoid early weaning.

## 1. Introduction

Persistent nipple pain is one of the most common reasons given by mothers for ceasing exclusive breastfeeding [1,2]. Breastfeeding pain has been expressed as a concern for mothers, not only in the immediate post-partum period, but for over one-third of mothers at two weeks and one month after birth [3,4]. It is not just painful, but can also cause psychological distress and interfere with general activity, mood, sleep, and bonding between mother and infant [5,6]. The most effective means of helping mothers to establish comfortable and painless breastfeeding in order to continue to breastfeed as long as they wish has yet to be established, and research is urgently needed in this area [7].

Nipple pain is often attributed to suboptimal positioning and attachment of the infant [8] although conclusive evidence is yet to be provided regarding which aspects(s) of positioning may be most important [9]. Other causes of nipple pain are: flat or inverted nipples [10,11], infant sucking action that causes nipple friction [11,12], infant ankyloglossia [13], infant palatal anomaly (bubble or high arched palate [14], strong infant suction [15,16], milk blisters (milk bleb or white spot) [12], infections (including *Candida albicans*, *Staphylococcus aureus*, *Herpes simplex* virus), psoriasis, dermatitis, and Raynaud’s phenomenon [12,17,18]. Insufficient milk supply may be a secondary effect of nipple pain as a result of inhibition of the milk ejection reflex due to pain [19], or to ineffective removal of milk as with an infant with ankyloglossia or cleft palate [20].

Correction of positioning and attachment is the most common experience-based recommendation for treatment of nipple pain, and when performed within the first week of birth has been associated with a longer duration of breastfeeding and fewer breastfeeding problems (including sore nipples) [21]. Improving the latch of infants four days to 12 months old (average 32 days) resolved breast pain in 65% of cases [22]. Correction of positioning and attachment with concurrent midwifery care was effective in reducing nipple pain and improving nipple condition within 20 days after birth [23]. However, other studies have shown that one, or even several, instruction sessions on proper positioning and attachment during the first few days after birth did not result in longer breastfeeding or fewer breastfeeding problems [24,25,26]. This highlights the fact that faulty positioning and attachment is not the only cause of nipple pain.

Nipple shields are suggested as a short-term measure for premature infants [27], or to keep an infant at the breast of a mother with inverted nipples [28] but caution is advised regarding long-term use [29].

It has been speculated that excessive nipple movement inside the infant’s mouth may be associated with nipple pain [30]. When there is frictional pain, highly purified anhydrous lanolin or vitamin A ointments may be used to alleviate the pain [1,31]. Frenotomy for ankyloglossia has been shown to be effective in relief of pain and increasing milk transfer [32,33]. There is little published evidence for the effect of palatal anomaly on breastfeeding, but in one case-study the pain was alleviated when the mother breastfed in a supine position [14]. Strong infant suction has been associated with nipple pain that has not been alleviated by advice from lactation consultants [15]. The suction is less strong when a nipple shield is used (McClellan, unpublished data). Clinical advice for milk blisters involves the application of a warm compress followed by breastfeeding or lancing the blister with a sterile needle [12]. Nipple pain due to infection can be treated with antibiotics for confirmed bacterial infection [34], or an antifungal for *Candida* infection [35]. Dermatitis can be treated by avoiding irritants and may require a short course of topical corticosteroid ointment [36]. Psoriasis of the nipple can also be treated with topical corticosteroids [37]. The pain due to Raynaud’s phenomenon can be treated by the use of simple measures such as warmth and avoidance of vasoconstrictors, as well as prescription of nifedipine [18].

There was insufficient evidence that glycerine gel dressings, lanolin with breast shells, lanolin alone, expressed breast milk, or all-purpose nipple ointment improved maternal perceptions of nipple pain in the first seven days of lactation [2] although highly purified anhydrous lanolin was found to be more effective than expressed breast milk over the first 14 days of lactation [31]. By contrast, it has been suggested that the use of lanolin may be associated with in increase in the rate of infection [38]. However, it is possible that this may have been the result of suboptimal hand hygiene.

In a Cochrane review, Dennis *et al.* [2] (page 2) have stated “It is unclear which intervention is the most appropriate treatment in the resolution or reduction of nipple pain. For health professionals to improve breastfeeding duration and exclusivity rates and to systematically address one of the most common difficulties encountered by breastfeeding mothers, a good understanding of nipple pain and corresponding treatment intervention is needed.”

### Aims

In order to provide corroborating evidence for the best strategies for the treatment of nipple pain that could prevent the cessation of exclusive breastfeeding, this study initially determined the frequency of nipple pain as a reason for presentation, the frequency of suspected infection as the cause of nipple pain, the results of nipple swabs that were cultured, the treatments prescribed and the effectiveness of the treatments at the Breast Feeding Centre of Western Australia (WA). We also identified the most common attributed aetiologies of nipple pain, the advice and/or treatment given to the mothers to alleviate the pain, and the effectiveness of the advice and treatment.

## 2. Experimental Section

Women who attended King Edward Memorial Hospital for their pregnancy and birth may make an appointment with a lactation consultant (IBCLC) at the Breastfeeding Centre of WA for assistance with breastfeeding difficulties. During the first visit the reason for consultation is recorded along with background information for the mother and infant and feeding behaviour of the infant. During a breastfeed the lactation consultant assesses the infant’s positioning and attachment and assesses if sucking action may be causing nipple friction. The amount of milk transferred during the breastfeed (by test-weighing) and, if appropriate, the amount of milk expressed after the breastfeed are measured. If there is concern about insufficient milk supply the mother is provided with BabyWeigh scales for measurement of a 24-h milk profile [39]. The lactation consultant also assesses the mother for flat or inverted nipples. If the nipple is red or pink, oedematous, sloughy, there are satellite lesions, or the pain is not resolved with correction of positioning and attachment and/or resting the nipples and expressing the breastmilk infection is suspected. Mid-stream milk samples from both breasts are hand expressed by the mother and sterile swabs moistened with sterile water are used to collect two swabs from each nipple. The milk and the swabs are sent to the microbiology laboratory for microscopy and culture and sensitivities are determined. The mother is also questioned regarding a history of Raynaud’s phenomenon, sensitivity of the nipples to cold, and bi- or tri-phasic colour changes associated with pain immediately after or between breastfeeds. The infant is assessed for orofacial anomaly, tongue protrusion, elevation, lateralization or bunching indicating ankyloglossia. Digital examination is used to detect posterior tongue tie, and to assess if the tongue can cup the finger. Palatal abnormalities (high arched palate or bubble palate) are assessed visually [40].

Based on these observations the lactation consultant makes a diagnosis and the mother is then provided with appropriate education and a plan for treatment. All mothers receive follow-up consultations during which the effectiveness of the treatment plan is assessed. Further education and plans for treatment are provided if necessary. Microbiology results are passed on the mother’s general practitioner or medical staff at King Edward Memorial Hospital. The general practitioner then provides the appropriate prescription.

All visits to the Breast Feeding Centre of WA were audited over two 6-month periods. For the first 6-month period retrospective data were collected from all mothers with suspected infection between March and August 2011, and for the second 6-month period, data were collected prospectively from all mothers who presented with nipple pain between January and June 2014. From the notes of the lactation consultant demographic and clinical variables were recorded, in addition to microbiology results when infection was suspected. The lactation consultants also followed up each visit with a telephone call. Recommended treatments and subsequent resolution of pain, or otherwise, were recorded.

In accordance with the National Statement on Ethical Conduct in Human Research the studies met the specific criteria as quality improvement audit projects and were approved by the Research Governance Office of the Women’s and Newborn Health Service (Quality Activity number 3984 approved 27 June 2012, Quality Activity number 5747 approved 22 February 2014).

## 3. Results

### 3.1. Suspected Infection as a Cause of Nipple Pain

During the first six-month audit there were 469 initial consultations. The median age of the mothers was 31 years (range 22–46 years). The median age of infants when the reason for consultation did not involve pain (*n* = 301) was 26 days (range 1–63 days). The median age of infants when the reason for consultation involved pain (*n* = 168, 36%) was 32 days (range 1–60 days).

Infection was suspected and swabs of the nipple(s) and milk samples were collected and cultured in 87 cases. Two cases were negative, and 32 grew only mixed skin flora. Advice and resolution of pain in these cases are presented in Table 1.

**Table 1:** Advice and resolution of pain when infection was suspected but cultures did not grow pathogenic organisms (*n* = 34).

*n*	Lactation Consultant Advice						Outcome		
	No Advice Recorded	Rest and Express	Nipple Shield	Cold Compress	Frenotomy	Domperidone	Pain Resolved or Resolving	Pain not Resolved	Lost to Follow-Up
**4**	x						2		2
**7**		x	x				5	1	1
**6**		x					2	2	2
**4**		x				x	2		2
**2**		x			x		1		1
**2**		x	x	x			2		
**1**		x	x					1	
**1**		x	x	x			1		
**1**		x		x			1		
**1**		x			x	x	1		
**1**		x	x	x		x		1	
**1**		x	x			x	1		
**1**		x	x		x	x	1		
**1**		x	x		x		1		
**1**					x				1

Infectious organisms were isolated in 53 cases. The most common organism detected was *Staphylococcus aureus* (47 cases), followed by *Candida albicans* (five cases) and *Streptococcus* spp. (four cases including two with *S. aureus*). One culture was positive for *S. aureus* and *Acinetobacter baumannii* and one culture was positive for *S. aureus* and *Pseudomonas stutzeri*) (Table 2).

Treatment for bacterial infections included oral antibiotics (penicillins, macrolides, cephalosporins, lincosamides) in 26 cases, and topical antibiotics (mupirocin and Kenacomb**^®^**—a proprietary product containing triamcinolone, neomycin, gramicidin and nystatin) in two cases, both oral and topical antibiotics in seven cases, oral antibiotics and antifungals in four cases, and oral and topical antibiotics and antifungals in one case). Pain was recorded as resolved or resolving (the pain was less than it was before treatment) in 27 of the treated cases (68%) after 1–65 days. Pain was not resolved in 10 cases after 5–74 days (median 14 days), and three cases were lost to follow-up. In nine cases, no prescribed pharmaceuticals were recorded, but management included advice to use cold compresses, rest the nipples and express breastmilk, use domperidone and/or frenotomy, or betadine (one case only). Pain was resolved or resolving in six cases at follow-up after 6–41 days. Pain was not resolved in three cases at follow-up after 5–50 days.

**Table 2:** Treatment prescribed and resolution of pain when infection was suspected and cultures grew pathogenic organisms (*n* = 53).

Organism(s)	*n*	Treatment									No Drugs/Not Recorded	Outcome		
		Oral				Topical		Antifungal						
		Penicillins	Macrolides	Cephalosporins	Lincosamide	Mupirocin	Kenacomb^®^	Fluconazole	Miconazole	Nystatin		Pain Resolved or Resolving	Pain not Resolved	Lost to Follow-up
*Staphylococcus*	15	x										9	5	1
	9										x	6	3	
	4			x								3		1
	3	x				x						2	1	
	2	x				x	x					2		
	2	x							x				1	1
	1	x				x				x		1		
	1				x	x						1		
	1					x						1		
	1	x					x						1	
	1						x					1		
	1				x								1	
	1		x									1		
*Streptococcus*	1	x										1		
	1			x								1		
*Staphylococcus + Pseudomonas*	1				x						x	1		
*Staphylococcus + Acinetobacter*	1				x							1		
*Staphylococcus + Streptococcus*	1	x							x				1	
	1			x								1		
*Staphylococcus + Candida*	1			x					x			1		
*Candida*	2							x	x			2		
	1							x				1		
	1										x		1	

Penicillins: flucloxacillin *n* = 20, dicloxacillin *n* = 3, amoxicillin *n* = 1, augmentin *n* = 1, unspecified penicillin *n* = 1.

Treatment prescribed for *C. albicans* (without bacterial infection) included fluconazole and/or miconazole or nystatin. In this practice, the treatment of choice is application of miconazole cream to nipples after each breastfeed [41] with concurrent application of miconazole gel to the infant’s oral cavity. If pain was not resolved with topical treatment, maternal oral fluconazole (150 mg once every second day for at least three doses) is recommended [35]. Nystatin was considered less effective and recommended for only one case in 2011 [42]. Pain was resolved or resolving in 3/5 cases after 6–22 days, while pain in the remaining case was not resolved.

During the second six-month audit, infection was suspected and swabs of the nipple(s) and milk samples were collected and cultured in 100 cases. The nipples of 85/100 cases were cracked, grazed, red or pink. One culture was negative, and 30 grew only mixed skin flora. Cultured organisms were: *S. aureus* alone (*n* = 52), *C. albicans* alone (*n* = 7), combinations of *S. aureus*, *C. albicans*, *Enterococcus faecalis* and/or coliform bacteria (*n* = 7), or other unspecified bacteria. Treatment prescribed for bacterial infections included oral and topical antibiotics in 42 cases. Pain was resolved or resolving in 29 cases (69%) at follow-up after 6–42 days. Antifungal treatment was prescribed for *C. albicans* (without bacterial infection) in six cases. Pain was resolved or resolving in three cases after 8–35 days, while pain in the remaining three cases were not resolved.

### 3.2. All Other Causes of Nipple Pain

During the second 6-month audit there were 708 initial consultations, of which 264 (36%) included nipple pain. Of these, 219 had introduced supplementary bottle or finger feeding, either with expressed breastmilk or supplementary infant formula. The median age of the mothers, when the reason for consultation involved pain, was 31 years (range 18–44 years) and the median age of infants was 10 days (range 2–134, plus one case when the mother suspected she had mastitis at 547 days).

The cause of the nipple pain was considered to be multifactorial in 89% of cases. Attributed causes of nipple pain were one or more of: incorrect positioning and attachment, ankyloglossia, infection, insufficient milk supply, mastitis, flat or inverted nipples, vasospasm, and palatal anomaly. In 234 cases there were between two and seven attributed causes of the nipple pain, in 65 different combinations, with frequencies of the different combinations ranging from 1 to 22 (Table 3). In 30 cases there was a single attributed cause of nipple pain. These were: positioning and attachment (*n* = 18), infection (*n* = 4), ankyloglossia (*n* = 3), insufficient milk supply (*n* = 2), palatal anomaly (*n* = 2), and flat or inverted nipples (*n* = 1). Ankyloglossia was diagnosed in 95 of the 117 cases with palatal anomaly.

The mothers received 2.1 ± 1.3 (range: 1–8) face-to-face consultations with a lactation consultant and 2.0 ± 1.4 (range: 0–9) follow-up telephone calls.

**Table 3:** Attributed causes of nipple pain (*n* = 264 cases).

Attributed Cause(s)	*n*	Attributed Cause(s)	*n*
pa.ank	22	pa.ank.ims.nip	2
pa.ank.hap	21	pa.ank.mas	2
pa.ank.ims.inf.hap	19	pa.ank.nip.inf	2
pa	18	pa.ank.nip.inf.hap	2
pa.inf	18	pa.ank.vas.hap	2
pa.ank.ims	12	ank.ims.hap	2
pa.ank.inf.hap	11	ank.inf.hap	2
pa.ank.ims.hap	10	ims.inf	1
pa.ank.ims.inf	10	nip	1
pa.ank.inf	10	pa.ims.nip.hap	1
pa.ims	7	pa.ims.nip.inf.hap	1
pa.ank.mas.inf	7	pa.ims.nip.vas.hap	1
inf	4	pa.nip.inf.hap	1
pa.hap	4	pa.nip.mas	1
pa.ims.hap	4	pa.nip.mas.inf	1
pa.ank.ims.nip.inf.hap	4	pa.ank.ims.inf.vas.hap	1
pa.ank.nip.hap	4	pa.ank.ims.mas.inf.vas	1
pa.ims.inf	3	pa.ank.ims.mas.vas.hap	1
pa.ims.inf.hap	3	pa.ank.ims.nip.inf	1
pa.inf.hap	3	pa.ank.ims.nip.inf.vas.hap	1
pa.nip	3	pa.ank.ims.vas.hap	1
pa.ank.ims.inf.vas	3	pa.ank.nip.mas.hap	1
pa.ank.ims.mas.hap	3	pa.ank.nip.vas	1
pa.ank.mas.inf.hap	3	ank.ims	1
ank	3	ank.ims.inf.hap	1
ims	2	ank.ims.mas	1
hap	2	ank.ims.mas.hap	1
pa.vas	2	ank.inf	1
pa.ims.nip.inf	2	ank.mas	1
pa.mas.inf	2	ank.mas.hap	1
pa.mas.inf.hap	2	ank.nip.hap	1
pa.ank.ims.mas.inf	2	ank.nip.inf.hap	1
pa.ank.ims.mas.inf.hap	2		

ank, ankyloglossia; hap, palatal anomaly (bubble or high arched palate); ims, insufficient milk supply; inf, infection; mas, mastitis; nip, flat or inverted nipples; pa, incorrect positioning and attachment; vas, vasospasm.

Recommendations for alleviation of nipple pain included management of the pain and treatment of the attributed cause(s). Management of the pain included resting the nipples by expressing breastmilk (*n* = 105), use of a nipple shield while breastfeeding (*n* = 135), use of cold (*n* = 9) or warm (*n* = 2) compresses on the nipples, and dressings (*n* = 4). Treatment of the pain included advice on correction of positioning and attachment (*n* = 241), frenotomy (*n* = 67), domperidone (*n* = 64), topical agents (medihoney, mupirocin, miconazole) (*n* = 67), oral antibiotics (*n* = 46), or antifungals (*n* = 17). There were 83 different suggested treatment combinations with only five of those combinations having a frequency of nine or more:

1. When incorrect positioning and attachment was the sole attributed cause (*n* = 18) all mothers received advice on correction of positioning and attachment. The pain was resolved or resolving in 8 of the 18 cases.
2. When incorrect positioning and attachment and ankyloglossia together were the attributed causes (*n* = 22) 16 mothers received advice on correction of positioning and attachment only and the pain was resolved or resolving in nine cases. Additionally, frenotomy was recommended for the other six infants, and the pain was resolved or resolving in five cases.
3. When incorrect positioning and attachment and infection together were the attributed causes (*n* = 18) infection was confirmed in 10 cases. All mothers received advice on correction of positioning and attachment. The pain was resolved or resolving for four of the six mothers who received advice on correction of positioning and attachment only, and the pain of three of the four mothers who were advised to use antibiotics was resolved or resolving.
4. When incorrect positioning and attachment, palatal anomaly and ankyloglossia together were the attributed causes (*n* = 21) all mothers received advice on correction of positioning and attachment. Frenotomy was recommended for eight infants, and the pain was resolved or resolving in four cases. Positioning and attachment, with or without pain management, was advised in 11 cases and the pain was resolved or resolving in seven of those cases. A further two mothers received advice to use an antifungal or other topical treatment and the pain was resolved or resolving in one case.
5. When incorrect positioning and attachment, palatal anomaly, ankyloglossia, infection and insufficent milk supply together were the attributed causes (*n* = 19) all mothers received advice on correction of positioning and attachment. Frenotomy was recommended for five infants, and the pain was resolved or resolving in four cases. Infection was confirmed in six cases, for which four were prescribed oral antibiotics, one mother was advised to use topical agents, and one received advice on management of the pain. In all but one case (prescribed antibiotics) the pain was resolved or resolving. Domperidone was advised in eight cases (one with frenotomy, four with treatment for infection, one with frenotomy and treatment for infection) and the pain was resolved in five cases.

Considering each attributed cause separately, the frequency of occurrence, targeted treatment and resolution of pain are presented in Table 4.

Nipple pain was resolved or resolving for 151 of the mothers (57%), and the median time taken for resolution of pain was 14 days (IQR 8, 23).

**Table 4:** Attributed causes, treatments and resolution of pain (*n* = 264 cases).

		Targeted Treatment			Pain Resolved or Resolving	
Attributed Cause	*n* *	Treatment	*n*	% of Cause	*n*	% of Treated
Incorrect positioning and attachment	238	Correction of positioning and attachment	238	100	137	58
Ankyloglossia	177	Frenotomy	62	35	43	69
		No frenotomy	115	65	64	56
Insufficient milk supply	104	Domperidone	62	60	35	56
		No domperidone	42	40	28	67
Palatal anomaly	117	Pain management	92	79	58	63
		No pain management	25	21	16	64
Flat or inverted nipples	32	Pain management	28	88	16	57
		No pain management	4	12	2	50
Vasospasm	14	Pain management	10	71	6	60
		No pain management	4	29	1	25
Mastitis	32	Antibiotics	12	38	8	67
		No antibiotics	20	62	15	75
Confirmed infection	69					
*C. albicans*	7	Antifungal	6	86	3	50
*S. aureus*	56	Oral antibiotics	18	32	12	67
		Oral plus topical antibiotics	14	25	10	71
		Topical antibiotics	8	14	6	75
		No antibiotics	16	29	7	44
Other bacteria	6	Oral antibiotics	2	33	1	50

* Total >264 as more than one attributed cause had been indicated for most cases; *S. aureus* includes 1 also with *C. albicans*, 2 with *Enterococcus*, and 1 with coliform bacteria; Other bacteria include *Enterococcus*, coliform or unspecified bacteria; Topical antibiotics include medihoney and mupirocin.

## 4. Discussion

During both six-month audits the incidence of nipple pain as a reason for consultation at the Breast Feeding Centre was 36% and during the second audit 83% of these mothers had already introduced the use of bottles or finger feeding. Although the mothers may have been expressing their breastmilk and the infants may still have been exclusively breastmilk fed, it illustrates how much the nipple pain interfered with feeding directly from the breast. These frequencies are consistent with the figures in the literature for breast pain as a common reason for women giving up exclusive breastfeeding [43]. Moreover, it has also been shown that persistent nipple pain also interferes with mood, sleep and general activity [5].

Well-known causes of nipple pain recorded during these six-month audits included suboptimal positioning and attachment, flat or inverted nipples, ankyloglossia, palatal anomaly, infections, Raynaud’s phenomenon (recorded as vasospasm), and insufficient milk supply. It is interesting that other less common published causes of nipple pain (strong infant suction, milk blisters, psoriasis, dermatitis) were not recorded. It is possible that there were no presentations of these skin conditions during the audit period, but it would be surprising if there were no cases of strong infant suction given that McCellan *et al.* [15] found that two thirds of mothers with chronic nipple pain had infants that exerted significantly stronger vacuums compared to infants of breastfeeding mothers without pain. This may not have been recorded as a cause because objective measurement of infant suction is currently only used as a research tool and is not yet part of routine clinical assessment [16]. Thus, clinical strategies are unlikely to be based on reduction of vacuums despite limited evidence suggesting nipple shields reduce vacuums back to those measured in control infants (McClellan, unpublished data). Objective measurement would allow for accurate diagnosis and monitoring of these cases.

Given the ubiquity of perception of the importance of correct positioning and attachment it is not unexpected that incorrect positioning and attachment was attributed as a contributing cause of nipple pain in 90% of cases. Although only one published study has demonstrated that correction of positioning and attachment is associated with fewer breastfeeding problems [21], it is known to be important to successful breastfeeding [44]. In this audit, resolution of pain after correction of positioning and attachment for 58% of cases supports this premise. This is not inconsistent with the published resolution rate of 65% after improvement of positioning and attachment [22]. The lack of improvement in 42% of cases suggests that either correction of positioning and attachment is not always effective and/or there are one or more contributing factors to the nipple pain as noted by the lactation consultants. Although Eglash *et al.* [45] quote Blair *et al.* [9] as a reference for incorrect latch being the cause of sore nipples, the latter study found only a non-significant trend relating the number of latching behaviours (rooting, gape, suck and seal) and the level of maternal pain. It is possible that the mothers in that study who had sore nipples also had, as in the current study, more than one factor contributing to their pain.

Ankyloglossia was the second-most common attributed cause of nipple pain, with a very high frequency (25% of initial consultations). However, this reflects only the frequency of ankyloglossia among mothers with breastfeeding concerns and not among the whole population, which is 3.2%–10.7% [46]. Although many infants with ankyloglossia are able to breastfeed effectively without causing nipple pain [13,47], it is now recognised that some of these infants have difficulty maintaining latch to the breast and do cause nipple pain [13]. In these cases, frenotomy can be effective in reducing pain and improving milk transfer [13,48]. In this audit frenotomy showed the equal-highest incidence of resolution of nipple pain. When frenotomy was not advised, this may have been due to the assessment of the lactation consultant that the ankyloglossia was not affecting the breastfeeding and not contributing to the nipple pain [47].

A high arched or bubble palate was the third-most common attributed cause of nipple pain. The shape of the palate is affected by continual pressure of the tongue against the palate *in utero* [49] and is therefore associated with ankyloglossia. Indeed, 95 (36%) of the infants in this audit were diagnosed with both ankyloglossia and palatal anomaly. The palatal anomaly by itself is thought to interfere with breastfeeding, and a change in breastfeeding position has been shown to improve milk transfer and decrease nipple pain [14]. In this audit, 106 cases where a palatal anomaly was recorded received correction of positioning and attachment and there was resolution of pain in 59% of cases whether or not advice for pain management was recorded.

In the cases in this audit, insufficient milk supply most likely occurred concurrently with nipple pain rather than being a cause of the nipple pain. The insufficient milk supply may be secondary to ineffective milk removal [50], or may be a result of low prolactin levels. In the latter case, the level of prolactin can be improved with the use of domperidone [51]. Its use is contraindicated if the mother has a pre-existing cardiac condition [52] or if there are potential adverse interactions with other medications. Although neither prolactin levels nor milk production were measured, the use of domperidone was associated with improvement of nipple pain in 56% of cases. Strong infant suction has been associated with nipple pain [15], and in the first month of lactation baseline suction pressure is stronger during non-nutritive sucking than during nutritive sucking [53]. Therefore, an increase in milk supply may lead to improved milk flow, weaker baseline suction pressure and diminished nipple pain. Improvement of nipple pain in 67% of cases where domperidone was not advised suggests that either insufficient milk supply was not the cause of the nipple pain, or correction of positioning and attachment improved milk removal and therefore milk supply. Indeed, resolution of pain when domperidone was advised may also have been due to the correction of positioning and attachment.

Suspected infection was confirmed for 61% of cases in the first six-month audit and 69% of cases in the second six-month audit. This frequency is similar to findings in Canada where 64% of cases with nipple damage had a positive culture [54]. Subsequent treatment with antibiotics during both audit periods resulted in resolution or improvement in 68%–69% of cases. This frequency is slightly lower than a smaller study in which pain was improved in 15 of 19 cases (79%) of confirmed *S. aureus* infection who were advised on optimal breastfeeding technique and use of oral antibiotics [34]. In the current audit we have no record of compliance when antibiotics were recommended, and this may contribute to the lower rate of resolution of pain in these cases. In addition, there was a wide range of antibiotics prescribed by the general practitioners and it is possible that the most efficacious one for each case was not chosen. There was resolution or improvement in pain for 67% of the cases of mastitis that were treated with antibiotics, but it is counterintuitive that there was improvement in pain for 75% of cases where antibiotics were not prescribed. Mastitis was never the sole attributed cause of nipple pain in this audit, and it may be that mastitis was not confirmed and was not, after all, contributing to the nipple pain. Overall, there was recorded resolution or improvement in nipple pain in 67% of cases after 14 days (IQR 7, 22) when confirmed infection was treated with antibiotics.

When treatment was advised for all causes of nipple pain during the second audit, there was recorded resolution or improvement in nipple pain in 57% of cases after 14 days (IQR 8, 23), and an average of two face-to-face consultations and two follow-up telephone calls. This frequency of improvement compares with 65% achieved for correction of positioning and attachment [22]. It is lower than recorded rates of 65%, 87% and 89% after 3, 7 and 14 days of treatment with HPA lanolin [31], but the mothers in that study had no anomalies in breast and/or nipple anatomy, chronic illness or other persistent pain-related conditions, which were included in the current study population. Unfortunately, we do not know if cases in which the pain was not resolved at last contact with the Breast Feeding Centre of WA subsequently found relief from the pain or whether they ceased breastfeeding.

Given the numerous combinations of attributed causes of nipple pain we suggest that nipple pain as presented at the Breast Feeding Centre of WA is often a result of a cascade of events. Suboptimal positioning and attachment (which could be due simply to positioning of the infant, or may be associated with ankyloglossia, palatal anomaly, maternal flat or inverted nipples) may result in unusually strong infant sucking vacuum, Raynaud’s phenomenon, friction of the nipple during sucking, and nipple trauma. The traumatised nipple is then vulnerable to infection. Strong infant sucking vacuum was not recorded as a possible cause of nipple pain, despite published studies showing that persistent nipple pain after correction of positioning and attachment was associated with strong infant sucking vacuum [15] and indicates the need for clinical measurement tools.

The numerous causes of pain and treatments available highlight the need for systematic diagnosis of the cause of pain, before a course of management is decided. This requires careful history taking and examination of mother and infant, as well as testing where appropriate and observation of a breastfeed. The results suggest that a greater emphasis on the education of mothers on correct positioning and attachment within the first week after birth to try to prevent nipple trauma and pain from occurring is necessary. Evidence from the literature indicates that one education session soon after birth is not sufficient [24,25,26]. Therefore, positioning and attachment may need to be assessed more than once during the first weeks and advice on correction of positioning and attachment may need to be repeated. In addition, the possible presence of ankyloglossia and palatal anomaly should be assessed as early as possible and intervention recommended as necessary. Indeed, when infants have ankyloglossia and feeding difficulties frenotomy in the first week of life has more benefit than when performed later [55].

When infection of the nipple is suspected, swabbing the nipples and sampling breastmilk enables treatment to be targeted, associated with at least two thirds of cases resolving [34]. However, other contributing factors that may have lead to the infection or added to the nipple pain also need to be addressed. Other objective measurements such as infant sucking vacuum [15] and milk production [39] could also lead to improved diagnosis and better targeted treatment of nipple pain.

This study in the local population underlines the complexity of diagnosis and treatment of nipple pain. The “Breast Pain Reasoning Model” [56] incorporates an assessment of the mother’s emotional state as well as the physical causes of nipple pain described here and elsewhere, and may assist in applying an holistic approach to this complex situation.

## 5. Conclusions

Nipple pain is a reason for consultation for 36% of mothers who present to the Breast Feeding Centre of WA. Over half of the mothers experienced improvement in their condition, but interpretation of the findings is complicated by the multiple aetiologies and combinations of treatments recommended.

The multiple attributed causes of nipple pain in breastfeeding mothers, possibly as a result of a cascade of events, suggests that effective early lactation management is crucial to avoid early weaning. Thorough antenatal breastfeeding education and correction of positioning and attachment in the first week after birth would assist in the prevention of nipple damage and subsequent infection. Early assessment and detection of ankyloglossia, suboptimal milk removal, strong infant suction and vasospasm would allow appropriate management to begin in a timely manner and prevent ongoing pain and psychological distress and possible compromise in milk supply in the longer term.
